# I Will Go a Fishing

**Published:** 1882-08

**Authors:** 


### "I WILL GO A FISHING."

To be sure. Not for money, but for rest and pleasure. The whole year is given over pretty much to the former, and now nature demands a change, if only for a few days. A friend writes, as a result of a year's close confinement: "I feel like saying, 'O, for a lodge in some vast wilderness,' where I might retire and take a long rest, away from the grinding of wheels on the streets, the screaming of locomotives, the jangling crash, worry and hubbub of business. 'Vanity of vanity! All is vanity.'" The writer of these words needs to make a strong and instant resolve to leave his work at once, and go not to "some vast wilderness" exactly, but to some quiet spot where are others like himself striving to make a complete change of habit, for the time being. Let it be as quiet as may be, but be sure that there is sufficient occupation for the mind to keep the thoughts from running in the old grooves. Books are well enough, but unless they, too, give new direction to one's moods, let them lie unopened. For these "few days" I am prescribing for the friend who cries out—" Vanity, vanity!" Walk, and talk if you can find a good listener, (not one who simply absorbs, but one who suggests) rise early in the morning, climb all the hills, cultivate every bit of curiosity lying dormant, that it may lead you to pry into every nook and cranny, so that even turning a corner will bring momentary gratification. Even come to "quizzing" the small boy one is sure to find, and you will be rewarded. He will tell you something you never guessed, and the pleasure he finds in telling it will suffuse itself through the air around. If only this quiet place includes a stream, or body of water and a boat, then will surely come for the noon-day siesta such real weariness as to make this sleep refreshing to body and mind. Only a few days of this "sweet nothing to do" will send the tired head and hand back to work with renewed energy and interest, for the *ennui* which is sure to be felt by the busy man in this short, aimless existence gives a real zest to the usual routine work.

